# Chest Computed Tomography Findings in Unilateral Pulmonary Fibrosis Secondary to Chronic Hypoperfusion

**DOI:** 10.1097/RTI.0000000000000764

**Published:** 2023-11-24

**Authors:** Cristina Marrocchio, Stephen M. Humphries, David A. Lynch

**Affiliations:** * Department of Medicine and Surgery, Unit of Radiological Sciences, University of Parma, Parma, Italy; † Department of Radiology, National Jewish Health, CO

**Keywords:** unilateral pulmonary fibrosis, fibrosing mediastinitis, absent pulmonary artery, high-resolution chest computed tomography

## Abstract

**Purpose:**

Unilateral lung fibrosis is uncommon and few cases secondary to parenchymal hypoperfusion have been reported, requiring further understanding of this entity. This study aims to report the chest computed tomography (CT) findings of patients with unilateral lung fibrosis related to parenchymal hypoperfusion observed in our institution.

**Patients and Methods:**

Patients with a chest CT between 2004 and 2022 showing a condition causing hypoperfusion of either lung and ipsilateral unilateral lung fibrosis were retrospectively identified. Clinical and scintigraphic data were collected. Pattern and distribution of fibrosis were recorded, and its progression was evaluated when follow-up was available. In adequate CTs, fibrosis was quantified using data-driven textural analysis (DTA). Affected and contralateral lungs and baseline and follow-up data were compared using the Wilcoxon signed-rank test.

**Results:**

Thirteen patients (male: 7, female: 6, median age: 61 y) were included; 5 with congenital unilateral absence of a pulmonary artery and 8 with fibrosing mediastinitis. The mean scintigraphic perfusion of affected lungs was 3.3% ± 1.1 compared with 96.7% ± 1.1 contralaterally (n = 7, *P* = 0.017). Fibrosis had a UIP pattern in one case, indeterminate in the others, and was most commonly diffuse craniocaudally and peripheral or central axially. DTA in 12 patients showed a mean fibrotic score of 32% ± 24.6 compared with 0.5% ± 0.4 in the contralateral lungs (*P* = 0.002). Median follow-up was 4.5 years (minimum to maximum: 1 to 13 y). Of 10 patients, fibrosis was progressive in 60%. DTA of 5 follow-up CTs showed increased reticulations (*P* = 0.043).

**Conclusion:**

In patients with lung hypoperfusion, the possible complication of lung fibrosis should be considered.

Although asymmetric fibrotic involvement of the lungs can be quite common, purely unilateral pulmonary fibrosis is a rare occurrence.^1^ Possible causes of unilateral or predominantly unilateral pulmonary fibrosis include radiation-induced and ventilator-induced lung injury, local chronic infection or inflammatory diseases, gastroesophageal reflux disease, and circulatory disorders.^2–5^ Specifically, some studies have described the occurrence of unilateral pulmonary fibrosis in patients with an underlying cause of hypoperfusion of the ipsilateral lung. Conditions that may cause such an asymmetric pulmonary vascularization are either congenital vascular anomalies (eg, unilateral absence or hypoplasia of a pulmonary artery), or acquired causes, which have been reported to be associated with lung fibrosis when chronic and long-standing.^6–17^ In one of these studies,^12^ the authors hypothesized that the development of fibrosis may be due to hypoperfusion, secondary to the pressure gradient between the systemic and the pulmonary arteries or high oxygen saturation level that may cause lung injuries, such as ischemia, infarction, bleeding, and inflammatory changes.

The current evidence on this specific type of pulmonary fibrosis remains limited to a few case reports or small case series and, to our knowledge, no study has specifically addressed this specific population of patients. Therefore, the understanding of this entity from a clinical and radiologic point of view remains unclear.

This study aims to retrospectively review cases of unilateral lung fibrosis related to lung hypoperfusion observed in our institution and describe the radiologic chest computed tomography (CT) findings in these patients.

## PATIENTS AND METHODS

This was a retrospective observational study, approved by the local Institutional Review Board. Informed consent of patients was waived.

Cases of unilateral hypoperfusion were prospectively recorded in a database by one of the authors from 2004 to 2022. The scans were then retrospectively reviewed. Patients were included if they were older than 18 years of age and had a chest CT scan between 2004 and 2022, showing a condition that caused unilateral hypoperfusion of the lung parenchyma and ipsilateral lung fibrosis. Subjects were excluded if they did not meet these inclusion criteria or had CT scans considered uninterpretable because of low image quality (eg, significant motion artifacts). Unilateral fibrosis was considered to be most likely related to parenchymal hypoperfusion based on the presence of parenchymal abnormalities that corresponded to the side of reduced vascularization and absent fibrosis in the contralateral side after other conditions associated with unilateral fibrosis (eg, history of prior infection or radiation) were excluded based on the clinical record.

Pertinent clinical data of patients were retrieved from the electronic medical record. Any available ventilation-perfusion scan and any chest CT in our institution or in other centers were considered. CT images were examined using a Siemens Syngo PACS system, at a standard lung window setting (window width: 1500, window level: −700), and mediastinal window setting (window width: 400, window level: 40). The CT scans were analyzed independently by a junior and a senior radiologist subspecialized in chest imaging with 1 year and more than 30 years of experience, respectively. Cases with discordant readings were subsequently reviewed jointly and a consensus was reached. Lung fibrosis was defined by the presence of any of the following findings:^18^ architectural distortion of the lung parenchyma, traction bronchiectasis, reticulations if associated with other signs of fibrosis, and honeycombing. Decreased lung volume was not used as a sole sign of fibrosis because of lung hypoplasia that can occur in the setting of hypoperfusion. The presence of other relevant parenchymal, pleural, or vascular findings was also noted. Ground-glass opacities were reported when not associated with other signs of venous congestion. If a follow-up CT scan was available before any eventual treatment, the progression of fibrotic abnormalities was assessed, and the pulmonary fibrosis was classified as stable or progressive based on the stability or worsening of the fibrotic changes, respectively.^19^

For baseline and follow-up images that met the required technical criteria, a quantitative assessment of lung fibrosis was carried out using data-driven textural analysis (DTA), as described elsewhere.^20^ If these scans were not technically adequate, the analysis was carried out on the earliest suitable CT, if any was available.

Categorical variables were reported as percentages, and continuous variables as median and range or mean and SD, as appropriate. Within patient analysis comparison of the affected lung and contralateral lung, and a comparison of variables at baseline and follow-up were done using the Wilcoxon signed-rank test. Patients with different causes of lung hypoperfusion were compared using the Mann-Whitney *U* test for continuous variables and the Fisher exact test or χ^2^ test for categorical ones. Data analysis was performed using SPSS Statistical Software (IBM Company).

## RESULTS

Twenty-two patients with a CT scan showing unilateral hypoperfusion were identified. Two cases were excluded because of the low quality of the CT images and 7 further patients were excluded because no signs of fibrosis were identified. Thirteen patients were finally included in the study. There were 7 males (54%) and 6 females (46%), of a median age at first evaluation in our institution of 61 years (minimum to maximum: 28 to 75). Five patients (38%) had a congenital absence of a pulmonary artery, and 8 (62%) had extrinsic unilateral pulmonary artery compression from fibrosing mediastinitis. The demographic and clinical characteristics of the included population are summarized in Table 1.

**TABLE 1:** Demographic and Clinical Characteristics of the Population

Characteristics of patients	Patients (n = 13); n (%)
Age (y)	
Median (minimum-maximum)	61 (28-75)
Sex	
Male	7 (54)
Female	6 (46)
Smoking history	
Current	0
Former	5 (38)
Never-smoker	8 (62)
Main pulmonary symptoms	
N available	12
Chest pain	2 (15)
Dyspnea	9 (69)
Recurrent respiratory infections	5 (38)
Hemoptysis	2 (15)
High-altitude pulmonary edema	1 (8)
Cause of lung hypoperfusion	
Fibrosing mediastinitis	8 (62)
Congenital absence of a main pulmonary artery	5 (38)

**FIGURE 1:**
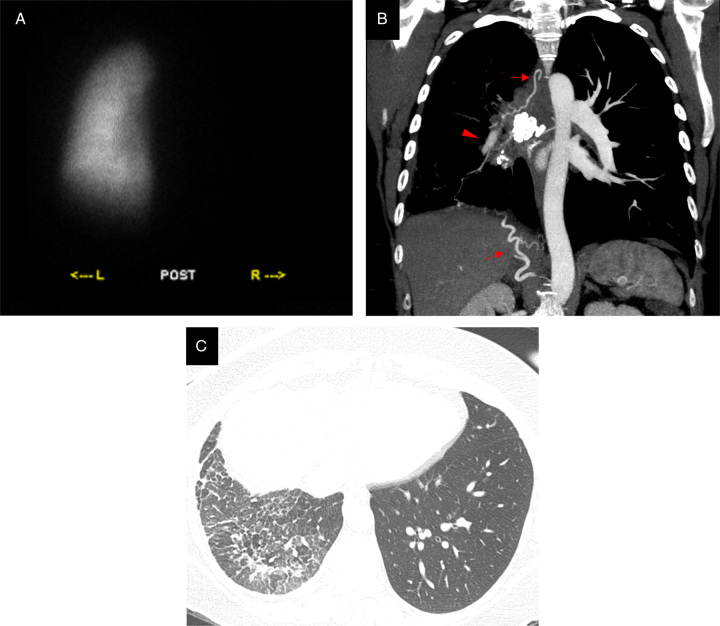
A 42-year-old woman with fibrosing mediastinitis. A, The perfusion scan shows no visible perfusion in the right lung, with quantified perfusion of 2% in the right lung, compared with 98% in the left one. Dynamic ventilatory images (not shown) demonstrated mildly decreased ventilation in the right lung on a single-breath image, which appeared to equilibrate throughout the washout phase. B, Coronal maximum intensity projection reconstruction of contrast-enhanced CT shows a mostly calcified mass in the right hilum compressing the ipsilateral pulmonary artery, which appears of reduced caliber and hypoattenuating (arrowhead). Hypertrophied collateral vessels can be recognized (arrows). C, Lung windows show unilateral right-sided irregular septal thickening and reticular abnormality, with bronchovascular distortion indicating fibrosis.

A ventilation-perfusion scan was available for 7 patients; ventilation data were missing in one case because this part of the examination was not performed during the coronavirus disease 2019 pandemic. All scans showed a marked unilateral reduction of lung perfusion, with mean perfusion of the affected parenchyma of 3.3% ±1.1, compared with 96.7% ±1.1 in the contralateral lungs (*P* = 0.017). The mean ventilation in the affected lungs was 33.3% ±11.4, compared with a mean ventilation in the contralateral lungs of 66.7% ±11.4 (*P* = 0.027; Fig. 1).

**FIGURE 2:**
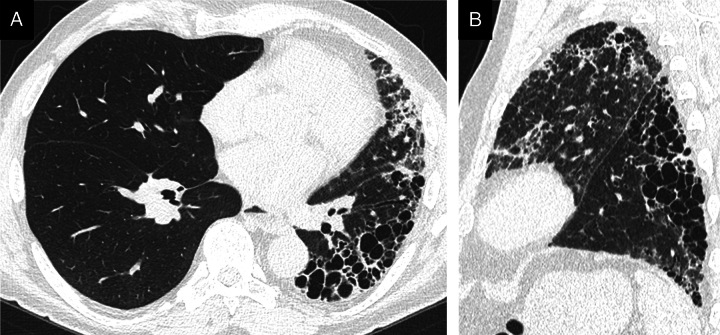
A 75-year-old man with fibrosing mediastinitis causing occlusion of the left pulmonary artery. A, The left lung shows volume loss and architectural distortion with extensive reticulations, traction bronchiectasis, and honeycombing. B, The pattern was classified as definite UIP, although no clear cranio-caudal gradient was recognized.

All patients had findings of lung fibrosis limited to one lung, as per inclusion criteria, specifically in the right lung in 7 cases (54%) and in the left one in 6 (46%). Architectural distortion was present in all patients, reticulations in 12 (92%), traction bronchiectasis in 10 (77%), and honeycombing in 6 (46%) cases. Ipsilateral volume loss was present in all but one patient who had fibrosing mediastinitis. The axial distribution of fibrosis was predominantly peripheral in 5, central in 5, and diffuse in 3 cases. Craniocaudally, it was most commonly diffuse (n = 7, 54%), followed by upper (n = 3), mid (n = 2), or lower (n = 1) lung predominant. Only one patient who had fibrosing mediastinitis had a pattern of definite UIP (Fig. 2), whereas the pattern was indeterminate in the other cases.

**FIGURE 3:**
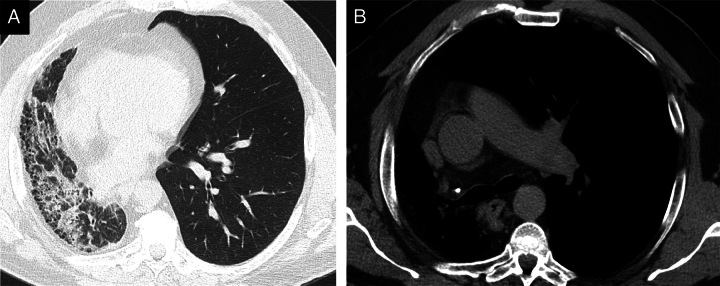
A 65-year-old man with right pulmonary artery agenesis. A, High-resolution CT shows extensive fibrotic changes and volume loss in the right lung, quantified to be 91.3% at DTA. B, The mediastinal window shows the absence of the right pulmonary artery.

The DTA analysis of lung fibrosis, ground-glass opacities, and lung volumes was carried out in 12 patients out of 13. Specifically, 10 patients had an adequate baseline CT scan and 7 patients an adequate CT at follow-up, with 5 cases having suitable CTs at both time points. The overall mean DTA fibrotic score in 12 patients was 32% ± 24.6 in the affected lungs, compared with 0.5% ±0.4 in the contralateral parenchyma (*P* = 0.002). Affected lungs had significantly more reticulation (*P* = 0.002) and honeycombing (*P* = 0.012). The affected lung volumes were also significantly reduced, with a mean of 1.5 L ± 0.7 compared with 3.4 L ± 0.8 contralaterally (*P* = 0.002), with some degree of volume loss identified in all patients (Table 2). When considering only the baseline scans, the mean fibrotic score of 10 patients was 30.8% ± 26.7 in the affected lungs compared with 0.4% ± 0.3 in the contralateral ones (*P* = 0.005; Fig. 3).

**TABLE 2:** DTA Quantitative Scores

Variable (%; mean, SD)	Affected lung (n = 12)	Contralateral lung (n = 12)
Fibrotic score	32±24.6	0.5±0.4
Reticulations	30.5±26.8	0.6±0.7
Honeycombing	2.8±5.3	0.009±0.1
Ground-glass opacities	5.9±3.3	3.9±0.9
Lung volume (L)	1.5±0.7	3.4±0.8

Other associated parenchymal abnormalities in the affected lungs included ground-glass opacities (n = 5), in one patient associated with septal thickening, and cystic abnormalities (n = 1). The contralateral lungs showed compensatory hyperinflation but no other relevant parenchymal findings. One patient with unilateral absence of pulmonary artery had associated right-sided aortic arch, lung abnormalities, and a focus of vascular dilation (presumed from systemic collaterals) in the peripheral affected lung. Pleural abnormalities were present in 5 out of 8 patients with fibrosing mediastinitis, ipsilateral to the affected lung. Contrast-enhanced CT in 10 patients showed hypertrophied collateral vessels from the bronchial, intercostal, internal thoracic, or phrenic arteries. The median pulmonary trunk diameter was 31 mm (minimum to maximum: 22 to 38 mm), with a diameter ≥30 mm in 9 patients, suggesting the presence of pulmonary hypertension. There were no statistically significant differences in demographic and imaging characteristics at baseline between patients with fibrosing mediastinitis and patients with unilateral absence of a main pulmonary artery.

**FIGURE 4:**
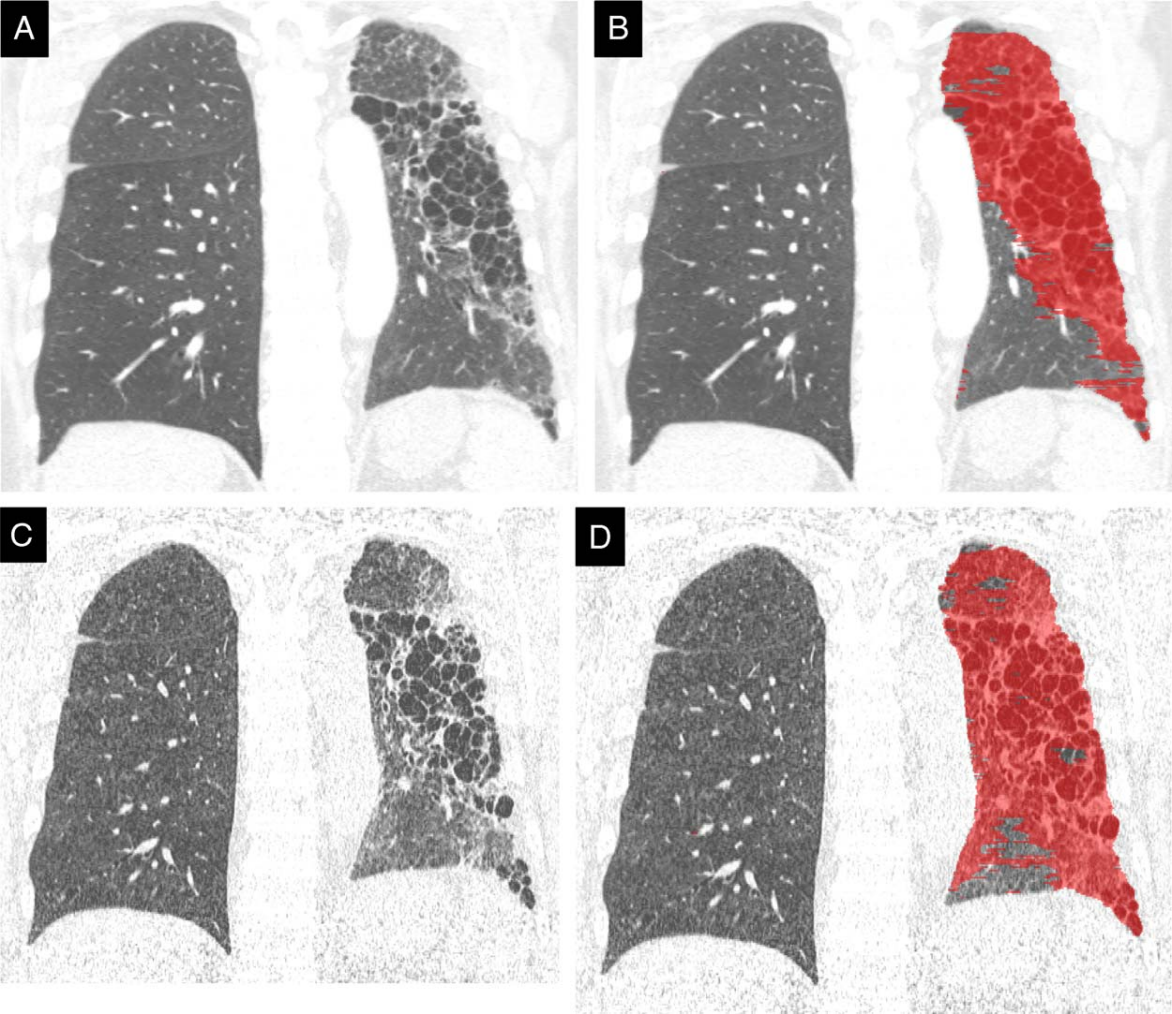
A 42-year-old woman with fibrosing mediastinitis, the same case shown in Figure 2. A and C, High-resolution CT shows fibrosis of the left lung in a UIP pattern at baseline (A) and at 2-year follow-up (C), with corresponding quantification of the fibrotic abnormalities (B and D). Fibrosis has progressed over 2 years, with increased reticulations and cystic abnormalities. DTA quantified a fibrotic score (color overlay) of 57.2% at baseline and 66.6% at follow-up; specifically, the percentage of reticular abnormalities was increased, and the lung volume was further reduced.

Clinical follow-up was available for 8 patients, ranging from 8 months to 13 years (median: 4 y). Two patients underwent pneumonectomy of the affected lung due to recurrent infections. Ten patients had an available follow-up CT, with a median follow-up time of 4.5 years (minimum to maximum: 1 to 13 y). In these, progression of the fibrotic abnormalities was observed in 6 cases (60%), with variable degrees and rapidity of progression of the parenchymal abnormalities. When comparing the baseline and follow-up quantitative scores of the affected lungs in the 5 patients for which adequate CTs were available at both timepoints, reticulations were significantly increased (*P* = 0.043; Fig. 4). No differences were noted in the contralateral lungs, specifically in their lung volumes. Fibrosis was particularly progressive in one patient whose fibrotic score increased from 57.2% to 66.6% at a 2-year follow-up. During follow-up, 3 patients had also worsened pleural abnormalities. The diameter of the pulmonary artery did not increase significantly (*P* = 0.066).

## DISCUSSION

In this retrospective study, the clinical and radiologic characteristics of a relatively large cohort of patients with unilateral lung hypoperfusion and associated unilateral ipsilateral lung fibrosis were described.

The reduction of perfusion in the affected lungs was confirmed by scintigraphy in 7 patients, and the observed decrease in perfusion was profound. Unilateral pulmonary lung fibrosis due to hypoperfusion has been reported secondary to both congenital and acquired, long-standing, conditions. Congenital abnormalities, and specifically congenital unilateral absence of a pulmonary artery, were present in 5 patients in our cohort and was an isolated abnormality except for one case. A recent meta-analysis reviewed the literature on isolated unilateral absence of pulmonary artery in adulthood from 1990 to 2016 and identified 65 reported cases, of which 6 (14%) had interstitial changes.^13^ Other congenital abnormalities, including unilateral hypoplasia of a pulmonary artery or absence of an interlobar pulmonary artery, have been reported to be associated with lung fibrosis in case reports.^7,12^ Less common is unilateral fibrosis in patients with acquired conditions causing chronic unilateral lung hypoperfusion. Case reports have described fibrotic abnormalities in patients with unilateral chronic pulmonary thromboembolism or a slow-growing sarcoma of the pulmonary artery.^8,10^ The most common cause of hypovascularity in our series was an underlying fibrosing mediastinitis compressing the main pulmonary artery. To the best of our knowledge, only one case of unilateral fibrosis secondary to fibrosing mediastinitis has been described.^9^ As this condition most commonly results from *Histoplasma capsulatum* infection, which is endemic in many regions of the United States,^21^ the epidemiological distribution of this disease may have favored the identification of these cases in our institution. No significant differences between patients having fibrosis related to a congenital absence of a pulmonary artery and fibrosing mediastinitis were observed in our study; however, the limited sample size prevents to draw final conclusions from this comparison.

When the pattern of fibrosis was specified in these cases of unilateral fibrosis reported in the literature, it was most commonly referred to as compatible with UIP.^8^ In our series, the distribution of fibrosis was quite variable both axially and craniocaudally, and, although 6 patients showed some honeycombing, only one patient had enough elements to define a UIP pattern, although lacking a clear craniocaudal gradient of the abnormalities. The fibrosis extent was quantified using DTA to have an estimate of lung involvement at baseline and to detect the progression of the fibrotic abnormalities in patients with adequate follow-up. In idiopathic pulmonary fibrosis, DTA quantification has been shown to correlate with semiquantitative visual scores and pulmonary function tests at baseline, with a moderate significant correlation with the degree of change in pulmonary function tests at follow-up.^20^ Therefore, lung fibrosis quantification at DTA is associated with lung function and can be an indication of disease severity.^20^ In our study, DTA confirmed the progression of the abnormalities, specifically in the extent of reticulations. The progression of parenchymal abnormalities is a key finding to recognize in patients with lung fibrosis, especially now that antifibrotic treatments are available. Although the follow-up in our patients was quite variable because of the retrospective nature of the study, a significant finding was the presence of progression of the abnormalities in 6 out of 10 patients with an available follow-up. However, the rapidity and degree of progression of the abnormalities were variable among patients. In one case with fibrosing mediastinitis, the abnormalities were already quite severe at baseline (57.2% fibrotic score at DTA) and had clear progression within 2 years of follow-up, increasing to 66.6%, underlining the importance of monitoring disease progression in these cases. In contrast, other patients showed only mild worsening, even at long-term follow-up. Moreover, the remaining 4 patients had stable disease, even after a long period of time, with a patient showing no progression even after 9 years. Therefore, further studies are necessary to assess whether there are any factors in this subpopulation of patients associated with the progression of fibrosis.

The mechanisms that determine the development of fibrosis from hypoperfusion of the lung parenchyma have not been clearly elucidated. Previous animal studies have shown that pulmonary artery ligation results in the development of systemic collateral vessels and neovascularization, with intense tissue remodeling at the visceral pleura.^22,23^ One study in rats with left pulmonary artery occlusion did show the development of organizing pneumonia with epithelial changes, inflammation, and fibrosis within 7 days after ligation.^24^ Arterial collaterals were identified in all our patients. These collaterals have been described in other studies as being often very hypertrophic and a possible cause of sometimes serious hemoptysis.^25,26^ Moreover, some authors hypothesized that these systemic-pulmonary anastomoses that develop in the setting of hypoperfusion may have a possible role in the pathogenesis of parenchymal fibrosis. As described before, Ryu et al^11,12^ hypothesized that a pressure gradient between the systemic and the pulmonary arteries or high oxygen saturation may induce lung injury resulting in cyst lung change. Ischemia and infarction due to thrombosis of collateral vessels may also be a factor. Hirosako et al,^10^ in their patient with chronic pulmonary thromboembolism, favored alveolar hemorrhage as the main cause of the fibrotic changes, due to the presence of cholesterol granulomas on histology.

This study has some limitations. The data collection was not systematic, and the true prevalence of fibrosis in individuals with pulmonary hypoperfusion cannot be estimated from this study. The sample size is small because of the rarity of the diseases causing unilateral lung hypoperfusion. Moreover, due to the retrospective design, patients did not have uniform follow-up and the technique of acquisition of the CT scans was variable, as many studies were acquired outside our institution. This may also have affected quantitative CT measurements. As our institution is a tertiary referral center for lung diseases and, specifically, interstitial lung diseases, the prevalence of patients with unilateral hypoperfusion and concomitant lung fibrosis in this study may not be reflective of the prevalence of fibrosis in the setting of hypovascularization in the general population.

In conclusion, this study describes a cohort of patients who developed unilateral lung fibrosis secondary to parenchymal hypovascularization. In patients with lung hypoperfusion, the possible complication of lung fibrosis should be considered.
